# Efficacy of GCWB106 (*Chrysanthemum zawadskii* var. *latilobum* extract) in osteoarthritis of the knee: A 12-week randomized, double-blind, placebo-controlled study

**DOI:** 10.1097/MD.0000000000026542

**Published:** 2021-07-02

**Authors:** Jeong Ku Ha, Jin Seong Kim, Joo Young Kim, Jong Bok Yun, Yun Young Kim, Kyu Sung Chung

**Affiliations:** a Department of Orthopedic Surgery and Sports Medical Center and Sports Medical Research Institute, Seoul Paik Hospital, College of Medicine, Inje University, Seoul, Republic of Korea; b GC Wellbeing Corporation, Seoul, Republic of Korea.

**Keywords:** *Chrysanthemum zawadskii* var. *latilobum*, knee, osteoarthritis, placebo

## Abstract

**Background:**

GreenCross Wellbeing Corporation (GCWB) 106 is a food item based on *Chrysanthemum zawadskii var. latilobum* extract. It has an inhibitory effect on joint inflammation.

**Objective:**

This study investigated the efficacy and safety of GCWB106 for osteoarthritis (OA) of the knee joint.

**Methods:**

Overall, 121 participants with mild OA were recruited and randomly divided into two groups. One group received GCWB106 for 12 weeks and the other group received placebo for 12 weeks. Outcomes were evaluated using the Korean-Western Ontario and McMaster Universities Index (K-WOMAC), visual analog scale, Korean Short Form Health Survey 36 score, and laboratory test results.

**Results:**

After 12 weeks of study treatment, the GCWB106 group exhibited a significant improvement compared with the placebo group in overall K-WOMAC score (*P* = .042) and K-WOMAC physical function score (*P* = .015). The GCWB106 group showed significant improvement in the visual analog scale pain score (*P* < .001) compared with the placebo group after 6 weeks and 12 weeks; no adverse drug reactions or serious adverse events were reported in either group.

**Conclusion:**

GCWB106 can safely reduce pain and improve knee function with therapeutic effects in OA of the knee joint.

**Level of evidence:**

Randomized, double-blind, placebo-controlled clinical study, Level I

## 1 Introduction

Osteoarthritis (OA) decreases osteoblast differentiation and increases the rate of cartilage degradation with increasing age in humans. Consequences of OA include loss of normal skeletal structure, cartilage damage, and ligament stiffness.^[1–3]^ The World Health Organization identifies OA as a disease that can cause mental issues, such as depression, helplessness, and alienation, as well as physical disability in daily life due to pain and functional disorders.^[4]^

Inflammation and pain are caused by bioactive substances such as prostaglandins and leukotrienes produced by cyclooxygenase or lipoxygenase using arachidonic acid as a substrate, cytokines (e.g., tumor necrosis factor [TNF]-α and interleukin [IL]-β), and free radicals (e.g., nitric oxide). The mechanisms of inflammation and pain reported to date are symptoms associated with cell damage resulting in the release of histamine and kinin, which cause vascular dilation, increased capillary permeability, and macrophage aggregation at inflammatory sites as well as edema, immune cell and antibody migration, pain, and fever.^[5]^

In severe cases of degenerative OA, surgical treatments including vitreous and synovial fluid removal using arthroscopy, synovial resection, curettage surgery, multiple perforation, corrective osteotomy, and artificial joint replacement are performed.^[6–8]^ In cases where the degenerative OA is not severe, drug therapy is typically administered.^[9]^ Drugs used for degenerative OA treatment include analgesics such as acetaminophen, nonsteroidal anti-inflammatory drugs (e.g., ibuprofen and indomethacin), and hyaluronic acid injected into joints and steroids. However, the treatment effect of these drugs is temporary and there are numerous side effects, such as hypersensitivity reactions, immune system deterioration, and adverse gastrointestinal events.^[10–12]^ Therefore, significant investigations continue for new OA treatment strategies that have more precise mechanisms and better safety.

GreenCross Wellbeing Corporation (GCWB) 106 is a newly formulated extract obtained from *Chrysanthemum zawadskii* var. *latilobum* (CZ), a perennial flowering plant belonging to the genus *Chrysanthemum* in the Asteraceae family. Conventionally, CZ has widely been used as an herbal medicine to relieve symptoms including hypertension and dizziness and to alleviate several inflammatory diseases such as stomatitis and colitis.^[13]^ CZ flower extracts have exhibited several pharmacological activities, including anticancer, anti-allergic, and anti-inflammatory effects.^[14–16]^ When applied to lipopolysaccharide -treated macrophages, CZ leaf extract inhibited inflammation by decreasing inflammatory mediator levels and inducing heme oxygenase-1.^[17]^ Recently, Gu et al suggested the therapeutic potential of CZ extract in inflammatory bone diseases based on its inhibition of the differentiation and formation of osteoclasts from bone marrow cells.^[18]^ Moreover, a CZ extract protected mice against rheumatoid arthritis via the suppression of nuclear factor-kappa-B mediated inflammation.^[19]^

Based on these observations, GCWB106 is expected to be protective and to improve mild OA symptoms in the knee. In addition, CZ extracts exert repressive effects on cytokines related to joint arthritis (e.g., TNF-α and IL-1β) and on inflammatory mediators (matrix metalloproteinase [MMP]-1, MMP-3, etc.) as well as inhibitory effects on osteoclast differentiation and bone resorption.^[19]^

Considering this background, the present study aimed to verify the effects of GCWB106 treatment on OA symptoms and the safety of its use compared with a placebo treatment. It was hypothesized that clinical results would be better for GCWB106 than for placebo in OA of the knee joint.

## 2 Patients and methods

### 2.1 Participants

Participants aged between 40 and 75 years with Kellgren and Lawrence classification for knee OA of grade I or grade II were enrolled; they were provided a baseline functional assessment of overall pain of at least 30 mm on a 100-mm visual analog scale (VAS).

Exclusion criteria were the as follows: participants diagnosed with arthritis by specific factors other than degenerative arthritis; those with a joint space of <2 mm; those diagnosed with severe arthritis with bony spur, irregular articular surface, osteocystoma, and other conditions; those experiencing cardiovascular, immune, infectious, and neoplastic diseases; those receiving treatment for gastritis and ulcers; those with uncontrolled hypertension with systolic blood pressure (SBP) of ≥160 mm Hg or diastolic blood pressure (DBP) of ≥100 mm Hg; those with fasting blood sugar of ≥180 mg/dL; those with thyroid stimulating hormone of <0.1 or >10 μIU/mL; those with alanine aminotransferase and aspartate aminotransferase levels more than three times the upper limit of normal; those with creatinine levels more than 3 times the upper limit of normal; pregnant or nursing women; those who use a diet of health functional foods and medicine to improve joint/cartilage health; those receiving treatment for mental disorders such as depression and schizophrenia; those using traditional pain-relieving therapies such as acupuncture, moxibustion, cupping therapy, and herbal medicine in the last 2 months; those participating in another human study within 2 months preceding this study; those with a history of allergy to the study materials; and those who were deemed by the principal investigator to be inappropriate for participation in this study because of a laboratory test result.

### 2.2 Preparation of GCWB106

GCWB106 was obtained from the GCWB (Seongnam-si, Korea). GCWB106 was extracted from the stems and leaves of CZ using 70% ethanol at 50°C and concentrated using a vacuum evaporator at 50°C. To prepare this as a powder, maltodextrin was added and the mixture was vacuum dried at 50°C. The extracted powder was sieved, followed by a standardization test to ensure the quality of the final raw sample. The end product was dispensed and packaged under the regulatory guideline for clinical study. Participants were instructed to consume 600 mg of GCWB106 (as 1 tablet/d) containing 250 mg of CZ extract or placebo (as 1 tablet/d) for 12 weeks. The main components of the placebo were 444 mg microcrystalline cellulose and 132 mg maltodextrin. The dose of CZ extract was calculated as 250 mg/d by calculating the human equivalent dose based on the effective dose of CZ extract confirmed in the non-clinical efficacy study (10–100 mg/kg).^[20]^ With respect to the active compound of CZ extract, linarin has recently been reported to be an active compound of CZ extract that exerts anti-OA effects, and the HPLC profiles of CZ extract and linarin content were reported.^[20]^

### 2.3 Clinical assessment

#### 2.3.1 Korean-Western Ontario and McMaster Universities Index (K-WOMAC) and VAS scores

The Western Ontario and McMaster Universities Osteoarthritis Index (WOMAC) has been used in research for over 25 years. The Index is available in >80 languages and measures three domains—pain, stiffness, and function—that are considered fundamental in the assessment of patient-reported outcomes in OA. The WOMAC has been recommended for use in clinical trials and been used in over 1000 peer-reviewed articles.^[21]^ Changes on the K-WOMAC scale were examined every 6 weeks from baseline to week 12. For K-WOMAC, the pain, stiffness, and physical function subscales and the total scores were assessed using a 5-point Likert scale, on which a decrease indicated an improvement in symptoms. VAS is a subjective assessment reported by the participant on a 10-cm horizontal line, where 0 indicates no pain and 10 indicates the worst pain. VAS is particularly useful for assessing changes in pain for individuals receiving therapy.

#### 2.3.2 Quality of life

Improvement of OA-related quality of life was examined by comparison to baseline using the Korean Short Form Health Survey 36 (KSF-36) after 6 weeks and 12 weeks of treatment. The KSF-36 questionnaire consisted of three categories (functioning, wellbeing, and overall health evaluation) with nine subdomains (physical function, social function, role physical, role emotional, mental health, vitality, bodily pain, general health, and health change), with each question rated on a 5-point Likert scale.

#### 2.3.3 Biochemical markers

The biochemical markers in the plasma, including MMP-2, MMP-3, MMP-9, MMP-13, cartilage oligomeric matrix protein (COMP), thiobarbituric acid-reactive substances (TBARS), and urinary C-terminal telopeptide of type II collagen (CTX-II), were measured from the baseline to 12 weeks. Blood and urine samples were obtained after overnight fasting. MMP-2, MMP-3, MMP-9, MMP-13, COMP, and TBARS levels were determined using a human enzyme-linked immunosorbent assay kit. The urinary CTX-II level was determined using a human CTX-II enzyme-linked immunosorbent assay kit. All analyses were performed at the same laboratory (GCCL, Yongin, Korea).

#### 2.3.4 Safety

Safety assessment involved conducting vital sign measurement (blood pressure, pulse, and body temperature), a routine battery of blood (complete blood cell count, differential white blood cell count, and aspartate aminotransferase, alanine aminotransferase, blood urea nitrogen, creatinine, albumin, total protein, and glucose levels) and urine tests, and adverse event monitoring.

### 2.4 Ethics statement

This study protocol was approved by the Institutional Review Board of Inje University Seoul Paik Hospital (PAIK 2017–09–004) and the Korean Clinical Research Information Service (number KCT0004238). All participants provided signed written informed consent for participating in the study.

### 2.5 Number of target participants

The number of study participants was determined as follows. A prior study regarding the efficacy of methylsulfonylmethane supplementation using the WOMAC scale showed that the mean change of WOMAC total score in the placebo group was 6.5 and that in the methylsulfonylmethane supplementation group was –8.4. The effect of GCWB106 was estimated to 10.3—approximately 70% of that of methylsulfonylmethane supplementation—and the standard deviation was estimated to 17.4, which was the maximum value in the reference text. A type I error rate of 5% and type II error rate of 20% were assumed based on the previous clinical trial focusing on the efficacy of methylsulfonylmethane supplementation in patients with OA. The number of participants required in each group of this study was calculated to be 45, considering the central limit theorem, which states that even if a population does not follow a normal distribution, if a sample size was 30 or higher, the distribution of the sample mean is approximated to normal distribution. Assuming the dropout rate for each group as 25%, overall, 120 participants would be required.

### 2.6 Statistical analysis

Data obtained from this clinical study were analyzed in three main forms: full analysis (FA), per protocol (PP), and safety set. The FA group composed of all participants randomly assigned to each group having at least 1 efficacy assessment following randomization. The PP group was defined as those in the FA group who completed this study without significant plan violations, and in this case, only the randomly assigned targets were excluded from the PP analysis. The safety set group included all randomized participants who were administered study products at least once. The significance of the difference between groups at each visit time was analyzed using an independent two-sample *t*-test or the Mann–Whitney *U*-test for continuous variables and the Pearson chi-squared test or Fisher exact test for categorical data. The significance of change within each group was analyzed using a paired *t*-test or the Wilcoxon signed rank test for continuous variables, and the McNemar test or McNemar exact test for categorical data. All statistical analyses were conducted with the SAS program, version 9.4 (SAS Institute, Inc., Cary, NC). A *P*-value of < .05 was considered significant.

## 3 Results

### 3.1 Inclusion

**Figure 1:**
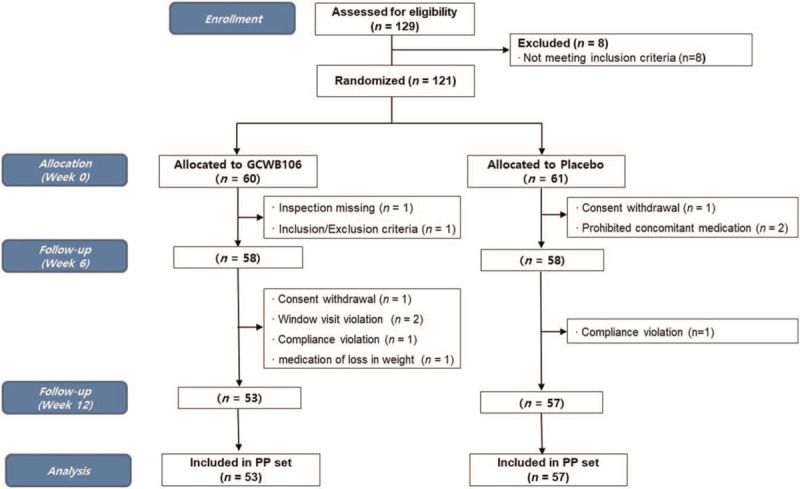
Flow chart of registered participants for the clinical trial (PP: per protocol).

In total, 129 volunteers were screened for selection suitability, and 121 participants were randomly assigned to a study group; 8 participants were eliminated by the selection/exclusion criteria and consent withdrawal. There were 60 participants in the GCWB106 group and 61 in the control group. Among these participants, 116 (58 in the GCWB106 group, 58 in the control group) continued to be a part of the study at 6 weeks, with the exceptions being individuals who withdrew consent or who upon inspection of their records were found to be taking a prohibited concomitant medication or in violation of the selection/exclusion criteria. During the trial, 6 more individuals were eliminated due to consent withdrawal, window visit violation, compliance violation, and medication affecting knee pain; finally, 110 participants (53 in the GCWB106 group, 57 in the control group) were included in the PP group (Fig. 1).

### 3.2 Baseline characteristics of study participants

The baseline characteristics of participants who completed the randomized controlled trial are described in Table 1. According to the sex of participants, 7 males (13.21%) and 46 females (86.79%) were included in the study group (GCWB106) and 6 males (10.53%) and 51 females (89.47%) were in the control group. A significant difference was observed between the groups (*P* = .663) based on the sex of the participant. Moreover, no significant difference was observed between the two groups in terms of age (*P* = .565). However, there were no significant differences between the groups in terms of SBP, DBP, drinking, smoking, exercise, X-ray (Kellgren and Lawrence grading scale), height, weight, and VAS score; thus, comparability between the groups was assumed.

**Table 1:** Baseline characteristics of two groups of subjects with mild osteoarthritis who participated in efficacy testing of GCWB106, an extract of *Chrysanthemum zawadskii* var. *latilobum*.

	GCWB106	Placebo	Total	
Characteristic	(Mean; SD)	(Mean; SD)	(Mean; SD)	*P*-value between Groups at Baseline
Gender (M: Male/F: Female)	53 (7M/46F)	57 (6M/51F)	110 (13M/97F)	.663
Age (year)	60.57 ± 8.93	59.63 ± 8.05	60.08 ± 8.46	.565
Height (cm)	156.40 ± 7.76	156.69 ± 6.35	156.55 ± 7.03	.831
Weight (kg)	60.01 ± 10.69	60.13 ± 8.57	60.07 ± 9.61	.947
SBP (mm Hg)	121.94 ± 10.89	125.77 ± 13.99	123.93 ± 12.69	.114
DBP (mm Hg)	73.11 ± 8.45	75.11 ± 8.23	74.15 ± 8.36	.213
Pain score on VAS (mm)	43.09 ± 8.32	42.86 ± 8.82	42.97 ± 8.54	.886
Kellgren and Lawrence grading scale, *n*(%)				
Grade 1	32 (60.38)	37 (64.91)	69 (62.73)	.623
Grade 2	21 (39.62)	20 (35.09)	41 (37.27)	

The values in the table are expressed as mean values (with standard deviation, SD). DBP = diastolic blood pressure, SBP = systolic blood pressure, VAS = visual analog scale.

### 3.3 K-WOMAC and VAS evaluation

The mean total score on the K-WOMAC scale at baseline was 28.13 ± 13.79 in the GCWB106 group and 24.96 ± 13.01 in the control group, with no significant difference between both groups. The improvement in K-WOMAC total score was –8.38 ± 11.99 in the GCWB106 group and –4.67 ± 14.98 in the control group after 12 weeks, and the difference in K-WOMAC total score improvement between both groups was significant (*P* = .042). The K-WOMAC total scores of both groups at the baseline, 6 weeks, and 12 weeks are shown in Table 2. In terms of the VAS score change, at both time points, the GCWB106 group showed a significant difference compared with the control group (*P* < .001). The VAS scores of both groups at the baseline, 6 weeks, and 12 weeks are shown in Table 3.

**Table 2:** Effect of 12 weeks of supplementation with GCWB106 on changes of the Korean-Western Ontario and McMaster Universities Osteoarthritis score in subjects with knee osteoarthritis.

		GCWB106	Placebo	*P*-value
		(n = 53)	(n = 57)	between Groups
		Mean ± SD	Mean ± SD	
Total score	Baseline (Visit 2)	28.13 ± 13.79	24.96 ± 13.01	.232
	6 weeks (Visit 3)	23.28 ± 14.96	21.91 ± 13.11	
	CFB	–4.85 ± 13.02	–3.05 ± 13.97	.252
	*P*-value^∗^	0.009	0.104	
	12 wk (Visit 4)	19.75 ± 13.86	20.30 ± 13.57	
	CFB	–8.38 ± 11.99	–4.67 ± 14.98	.042
	*P*-value^∗^	*P* < .001	0.022	
Pain	Baseline (Visit 2)	5.38 ± 2.81	4.96 ± 2.73	.390
	6 wk (Visit 3)	4.55 ± 3.19	4.16 ± 2.97	
	CFB	–0.83 ± 3.16	–0.81 ± 3.40	.951
	*P*-value^∗^	0.061	0.078	
	12 wk (Visit 4)	3.60 ± 3.01	3.93 ± 2.84	
	CFB	–1.77 ± 3.00	–1.04 ± 3.33	.226
	*P*-value^∗^	*P* < .001	0.022	
Stiffness	Baseline (Visit 2)	2.58 ± 1.46	2.61 ± 1.47	.970
	6 wk (Visit 3)	2.04 ± 1.44	2.12 ± 1.48	
	CFB	–0.55 ± 1.73	–0.49 ± 1.50	.733
	*P*-value^∗^	0.025	0.016	
	12 wk (Visit 4)	1.91 ± 1.40	2.07 ± 1.39	
	CFB	–0.68 ± 1.53	–0.54 ± 1.64	.585
	*P*-value^∗^	0.002	0.015	
Physical function	Baseline (Visit 2)	20.17 ± 10.24	17.39 ± 9.63	.116
	6 wk (Visit 3)	16.70 ± 11.18	15.63 ± 9.39	
	CFB	–3.47 ± 9.57	–1.75 ± 10.13	.118
	*P*-value^∗^	0.010	0.196	
	12 wk (Visit 4)	14.25 ± 10.06	14.30 ± 9.95	
	CFB	–5.92 ± 8.72	–3.09 ± 11.28	.015
	*P*-value^∗^	*P* < .001	0.043	

The values in the table are expressed as mean values (with standard deviation, SD). CFB, changes from baseline, K-WOMAC, Korean-Western Ontario and McMaster Universities Osteoarthritis Index, SD = standard deviation.

∗ Derived from paired *t*-tests performed for values obtained at baseline and after the trial.

**Table 3:** Effect of 12 weeks of supplementation with GCWB106 on changes of the Visual Analog Scale score in subjects with knee osteoarthritis.

		GCWB106	Placebo	*P*-value
		(n = 53)	(n = 57)	between Groups
		Mean ± SD	Mean ± SD	
VAS	Baseline (Visit 2)	43.09 ± 8.32	42.86 ± 8.82	.935
(mm)	6 wk (Visit 3)	34.15 ± 10.07	39.21 ± 8.34	
	CFB	−8.94 ± 7.96	−3.65 ± 4.87	*P *< .001
	*P*-value^∗^	*P* < .001	*p* < 0.001	
	12 wk (Visit 4)	30.30 ± 9.92	36.77 ± 9.07	
	CFB	−12.79 ± 10.97	−6.09 ± 6.95	*P *< .001
	*P*-value^∗^	*P *< .001	*P* < .001	

The values in the table are expressed as mean values (with standard deviation, SD).

∗ Derived from paired *t*-tests performed for values obtained at baseline and after the trial. CFB = changes from baseline, VAS = visual analog scale.

### 3.4 Quality of life

The analysis of improvements in individual symptoms of KSF-36 showed that 10 symptoms had significantly improved after GCWB106 administration, whereas two symptoms had improved in the placebo group. As shown in Table 4, the GCWB106 group demonstrated a significant difference compared with the placebo group in terms of bodily pain (*P* < .031) at 6 weeks as well as in emotional role (*P* = .041) and wellbeing (*P* = .038) at 12 weeks.

**Table 4:** Effect of 12 weeks of supplementation with GCWB106 on changes of the Korean short form health survey-36 score in subjects with knee osteoarthritis.

		Baseline	6 wk	12 wk
Variables	Treatment	Mean ± SD	Mean ± SD	Mean ± SD
Functioning	Placebo	69.14 ± 15.06	72.74 ± 14.29^∗^	72.15 ± 16.30
	GCWB106	66.31 ± 14.14	70.22 ± 15.57^∗^	71.43 ± 14.32^∗^
Physical functioning	Placebo	59.39 ± 19.18	60.88 ± 17.50	63.51 ± 19.48
	GCWB106	58.49 ± 18.57	58.30 ± 19.19	59.25 ± 19.84
Social functioning	Placebo	79.39 ± 15.22	84.65 ± 15.31^∗^	83.11 ± 14.85
	GCWB106	75.94 ± 17.82	82.55 ± 14.78^∗^	83.96 ± 14.99^∗^
Role–physical	Placebo	72.15 ± 20.83	77.08 ± 19.71	74.89 ± 19.78
	GCWB106	68.99 ± 17.51	73.94 ± 22.63	75.00 ± 20.03^∗^
Role–emotional	Placebo	74.56 ± 20.74	78.80 ± 18.10	75.58 ± 21.70
	GCWB106	69.34 ± 20.20	76.89 ± 15.98^∗^	78.62 ± 16.95^∗^^,^^†^
Well-being	Placebo	59.76 ± 13.53	62.85 ± 13.35	61.14 ± 14.62
	GCWB106	57.51 ± 11.87	62.18 ± 12.14^∗^	63.37 ± 13.07^∗^^,^^†^
Mental health	Placebo	64.30 ± 16.46	66.84 ± 15.80	63.86 ± 17.17
	GCWB106	62.55 ± 14.86	66.04 ± 13.85^∗^	67.17 ± 14.76^∗^
Vitality	Placebo	55.37 ± 15.82	60.09 ± 15.34^∗^	55.70 ± 16.59
	GCWB106	53.54 ± 15.72	57.08 ± 15.70	58.02 ± 16.19
Bodily pain	Placebo	57.72 ± 15.36	59.30 ± 15.68	64.39 ± 14.76^∗^
	GCWB106	53.77 ± 17.01	62.64 ± 18.31^∗^^,^^†^	64.34 ± 18.14^∗^
Overall health evaluation	Placebo	49.12 ± 15.70	51.97 ± 14.75	51.68 ± 15.10
	GCWB106	48.43 ± 15.34	53.22 ± 15.96^∗^	56.21 ± 16.34^∗^
General health	Placebo	50.18 ± 17.11	52.72 ± 16.45	51.67 ± 16.78
	GCWB106	49.72 ± 17.36	54.15 ± 17.59^∗^	56.89 ± 17.95^∗^
Health change	Placebo	43.86 ± 19.64	48.25 ± 15.57	51.75 ± 17.59^∗^
	GCWB106	41.98 ± 17.52	48.58 ± 20.46^∗^	52.83 ± 20.60^∗^

The values in the table are expressed as mean values (with standard deviation, SD). KSF-36 = Korean Short Form Health Survey-36.

∗ Derived from paired *t*-tests performed for values obtained at baseline and after the trial.

† Derived from Wilcoxon rank sum test compared between groups.

### 3.5 Biochemical markers

Analysis of biochemical markers showed a decrease in MMP-3 and urine CTX-II in the GCWB106 group compared with the baseline; however, this difference was not significant (*P* = .471 and *P* = .814) compared with the placebo group. TBARS, an antioxidant biomarker, showed a decrease in the GCWB106 group, but no significant difference was observed between the 2 groups (*P* = .480) (data not shown).

### 3.6 Investigator evaluation

The investigator conducted an evaluation of improvement at 6 and 12 weeks of treatment. The overall symptoms showed an improvement in the GCWB106 group compared with that observed in the control group. A significant difference was observed between groups after 6 weeks of treatment (*P* = .001). In addition, there was a significant difference between groups even after 12 weeks of treatment (*P* = .003) (Table 5).

**Table 5:** Effect of 12 weeks of supplementation with GCWB106 on the investigator assessment of improvement in subjects with knee osteoarthritis.

	GCWB106	Placebo	*P*-value
	(n = 53)	(n = 57)	between Groups
	Mean ± SD	Mean ± SD	
6 wk (Visit 3)	2.36 ± 0.52	2.67 ± 0.48	.001
12 wk (Visit 4)	2.28 ± 0.63	2.56 ± 0.50	.003

The values in the table are expressed as mean values (with standard deviation, SD).

### 3.7 Safety

Adverse events were reported in 5 cases in 3 patients in the GCWB106 group (5.00%) and in 5 cases in 4 patients in the placebo group (6.56%) (Table 6). No significant difference was noted in frequency between both groups (*P* = 1.000). Among the adverse events reported, adverse drug reactions and serious adverse events were not observed in either group.

**Table 6:** Adverse events occurring during the study.

	GCWB106	Placebo	Total
Adverse experiences (n, %)	(n = 60)	(n = 61)	(N = 121)
Eye disorders	2 (3.33)	2 (3.28)	4 (3.31)
Conjunctival cyst	1 (1.67)	0 (0.00)	1 (0.83)
Dacryostenosis acquired	0 (0.00)	1 (1.64)	1 (0.83)
Dry eye	1 (1.67)	1 (1.64)	2 (1.65)
Gastrointestinal disorders	2 (3.33)	0 (0.00)	2 (1.65)
Nausea	1 (1.67)	0 (0.00)	1 (0.83)
Dyspepsia	1 (1.67)	0 (0.00)	1 (0.83)
Parotid gland enlargement	1 (1.67)	0 (0.00)	1 (0.83)
Musculoskeletal and connective tissue disorders	0 (0.00)	1 (1.64)	1 (0.83)
Musculoskeletal pain	0 (0.00)	1 (1.64)	1 (0.83)
Skin and subcutaneous tissue disorders	0 (0.00)	2 (3.28)	2 (1.65)
Acne	0 (0.00)	1 (1.64)	1 (0.83)
Eczema	0 (0.00)	1 (1.64)	1 (0.83)
Total patients with adverse events	3 (5.00)	4 (6.56)	7 (5.79)
Total of ADR^∗^	0 (0.00)	0 (0.00)	0 (0.00)
Total of SAE	0 (0.00)	0 (0.00)	0 (0.00)

∗ ADR, adverse effects reported and rated by the investigator as possibly related, probably related, and definitely related. ADR = adverse drug reactions, SAE = serious adverse events.

Regarding the analysis of vital signs, changes in SBP and DBP within the GCWB106 group showed significant differences but were considered to be within the normal range with no clinical implications. In the hematological and biochemical analyses, significant differences in total cholesterol and triglyceride levels were observed between the study and control groups at the time of screening. There were significant differences in the changes in creatinine levels in the GCWB106 group and changes in hemoglobin, Na, creatinine, and uric acid levels in the control group. However, the changes observed were within the normal range and were considered to have no clinical implications.

## 4 Discussion

The present study aimed to verify the validity and safety of GCWB106, a food with health functions, based on *C zawadskii* var. *latilobum* extract, in participants with mild knee OA. The study findings revealed that when participants with mild knee OA were treated with GCWB106 for 12 weeks, they showed a significant improvement in the total score and physical function score on the K-WOMAC test and on the VAS for pain compared with a placebo-treated control group. Although the improvements observed in the pain and stiffness scores of the K-WOMAC were not significant, the pain and stiffness scores decreased after 12 weeks in the study group. Therefore, the present study demonstrates that compared with placebo treatment, GCWB106 showed a higher ability to reduce pain and improve physical function, quality of life, and joint health.

CZ has routinely been used as a traditional remedy for several inflammatory diseases, and the mechanisms for its anti-inflammatory effects have been investigated.^[22]^ Some studies have reported on benefits of CZ extracts such as suppression of the arthritis index in the blood and inhibition of cartilage tissue destruction and gene expression related to arthritis; however, most studies have demonstrated efficacy in animal models. Moreover, our preliminary study verified the cytokines (TNF-α, IL-1β) and inflammatory mediators (MMP-1, MMP-3) associated with joint inflammation in animal models. Therefore, this study was conducted to verify the effect and safety of CZ extracts as treatments in humans with OA.

For the study, participants with mild degenerative arthritis symptoms having a VAS pain score exceeding 30 mm and exhibiting grade I and II of the Kellgren and Lawrence grading scale were recruited; patients with joint space of >2 mm as determined by X-ray and those with moderate arthritis were excluded. Thus, the present study focused on the efficacy of CZ treatment in early OA. For the investigation of the safety aspects of this natural substance, individuals experiencing potential side effects of nonsteroidal anti-inflammatory drugs in the gastrointestinal system, particularly gastritis and gastric ulcer^[23]^, were excluded during screening, and participants who injected drugs for weight loss were excluded from data analysis because these drugs could have an indirect effect on results of the pain questionnaire.^[24]^ Participants were randomly assigned in a double-blind fashion; accordingly, the investigators, participants, and examiners were blinded during the study. Blinding was maintained until the last visit, and no issues with double blinding occurred during the study period. Compliance was over 90% in both groups, thereby increasing the reliability of this study.

Psychological improvements and improved social wellbeing may be important treatment goals in some patients with OA because of related factors such as sleep disorders, loneliness, and mood disorders accompanying OA pain.^[25]^ K-WOMAC was mainly developed to evaluate patients with arthritis of the lower extremity, whereas KSF-36 is a tool developed to understand a person's overall condition.^[26]^ Previous studies have shown that KSF-36 assesses an important effect in the quality of life of patients with OA.^[27]^ The present study demonstrated an improvement of OA-related quality of life using the KSF-36 questionnaire after 6 and 12 weeks of treatment compared with the baseline. There were significant improvements in functioning, wellbeing, and overall health evaluation in the GCWB106 group treated for 12 weeks. Moreover, a significant improvement was comprehensively confirmed in the nine subdomains compared with the baseline. In addition, compared with the control group, it confirmed that the emotional role and wellbeing improved across 12 weeks. This confirmed that GCWB106 could improve aspects of the participants’ health related to quality of life, according to the KSF-36 questionnaire.

In joints, articular cartilage covers the bone surface and reduces friction that can occur when the joint components move against each other. Articular cartilage includes chondrocytes albeit very few, and almost all articular cartilage (98%–99%) consists of a cartilage matrix. The main components of the cartilage matrix are type II collagen, proteoglycan, water, and glycoproteins.^[28]^ In OA, these components are degraded by several causes. Among the proteinases that degrade cartilage collagens in joint disease, MMPs have received the most attention because they degrade native collagens and proteoglycans. MMPs are zinc-dependent endopeptidases, including collagenases (MMP-1, MMP-8, and MMP-13), gelatinases (MMP-2 and MMP-9), and stromelysin-1 (MMP-3).^[29]^ In addition, COMP is primarily secreted from chondrocytes and synovial cells and is an indicator mainly reflecting the metabolic state of cartilage.^[30]^ However, in the present study, these biomarkers were not significantly different among the groups. There is thus a need for a clinical trial including individuals with knee OA of further severity for obtaining elucidated results for collagen-related biomarkers.

There was no safety issue with GCWB106. In the present study, adverse events were reported in 10 cases (n = 7). Among the adverse events, adverse drug reactions and serious adverse events were not reported in either group. In the analysis of vital signs, changes in SBP and DBP within the GCWB106 group showed significant differences but were considered to be within the normal range, thereby having no clinical implications. In the hematological and biochemical analyses, significant differences in total cholesterol and triglyceride levels were observed between the GCWB106 and control groups when observed at the time of screening. Furthermore, significant differences were observed in changes in creatinine levels in the GCWB106 group and changes in hemoglobin, Na, creatinine, and uric acid levels in the control group. However, these changes were within the normal range and were considered to have no clinical implications. The study confirmed that ingestion of GCWB106 was safe.

There are limitations of this trial worth considering. First, considering that the participants in this study were restricted to volunteers, it was difficult to represent the general population with the small sample size. Second, because the study was conducted on individuals with mild OA having Kellgren and Lawrence grade measurements ranging between I and II, there was difficulty in identifying some of the results obtained in the clinical trial and in generating statistically meaningful data for biochemical markers. Therefore, to produce more statistically meaningful data, a study in participants with severe OA is a recommended next step. Third, in this study, the significance of the difference between both groups at each visit time was analyzed using independent t-tests or Mann–Whitney tests rather than ANOVA or MMRM, Therefore, there may be concerns that the effects of GCWB106 presents validation of the effect of repeated measurements. However, the sample size of this study was calculated considering this issue. Therefore, the authors believe that compared with placebo, the evidence for the efficacy of GCWB10 for the improvement of OA symptoms is well supported by the findings.

## 5 Conclusions

The present study demonstrates that GCWB106, when compared with placebo treatment, shows better ability to reduce pain and to improve physical function, quality of life, and joint health in patients with early OA. Therefore, GCWB106 can be recommended as a good option in the management of patients with early OA.

## Author contributions

K. S. Chung, J. K. Ha, and J. Y. Kim equally contributed to the conception and design of the research; Y. Y. Kim and J. S. Kim contributed to the acquisition and analysis of the data; J. B. Yun contributed to the interpretation of the data; and K. S. Chung and J. K. Ha drafted the manuscript. All authors critically revised the manuscript, agree to be fully accountable for ensuring the integrity and accuracy of the work, and read and approved the final manuscript.

**Conceptualization:** Jeong Ku Ha, Joo Young Kim, Kyu Sung Chung.

**Data curation:** Jong Bok Yun.

**Formal analysis:** Yun Young Kim.

**Investigation:** Yun Young Kim, Jin Seong Kim.

**Methodology:** Jeong Ku Ha, Joo Young Kim, Kyu Sung Chung.

**Supervision:** Kyu Sung Chung.

**Writing – original draft:** Jeong Ku Ha.

**Writing – review & editing:** Jin Seong Kim, Kyu Sung Chung.
